# Metabolite profiling reveals the interaction of chitin-glucan with the gut microbiota

**DOI:** 10.1080/19490976.2020.1810530

**Published:** 2020-09-06

**Authors:** Julie Rodriguez, Audrey M. Neyrinck, Zhengxiao Zhang, Benjamin Seethaler, Julie-Anne Nazare, Cándido Robles Sánchez, Martin Roumain, Giulio G. Muccioli, Laure B. Bindels, Patrice D. Cani, Véronique Maquet, Martine Laville, Stephan C. Bischoff, Jens Walter, Nathalie M. Delzenne

**Affiliations:** a Metabolism and Nutrition Research Group, Louvain Drug Research Institute, UCLouvain, Université Catholique de Louvain, Brussels, Belgium; b Department of Medicine, University of Alberta, Edmonton, Canada; c Institute of Nutritional Medicine, University of Hohenheim, Stuttgart, Germany; d Rhône-Alpes Research Center for Human Nutrition, Université-Lyon, CarMeN Laboratory, Hospices Civils de Lyon, Lyon, France; e Bioanalysis and Pharmacology of Bioactive Lipids Research Group, Louvain Drug Research Institute, UCLouvain, Université Catholique de Louvain, Brussels, Belgium; f WELBIO- Walloon Excellence in Life Sciences and BIOtechnology, UCLouvain, Université Catholique de Louvain, Brussels, Belgium; g KitoZyme, Parc Industriel des Hauts-Sart, Herstal, Belgium; h Department of Agricultural, Food & Nutritional Science and Department of Biological Sciences, University of Alberta, Edmonton, Canada; i APC Microbiome Ireland, Department of Medicine, and School of Microbiology, University College Cork, Cork, Ireland

**Keywords:** Gut microbiota, chitin-glucan, fiber, SCFA, bile acids

## Abstract

Dietary fibers are considered beneficial nutrients for health. Current data suggest that their interaction with the gut microbiota largely contributes to their physiological effects. In this context, chitin-glucan (CG) improves metabolic disorders associated with obesity in mice, but its effect on gut microbiota has never been evaluated in humans. This study explores the effect of a 3-week intervention with CG supplementation in healthy individuals on gut microbiota composition and bacterial metabolites. CG was given to healthy volunteers (n = 15) for three weeks as a supplement (4.5 g/day). Food diary, visual analog and Bristol stool form scales and a “quality of life” survey were analyzed. Among gut microbiota-derived metabolites, bile acids (BA), long- and short-chain fatty acids (LCFA, SCFA) profiling were assessed in stool samples. The gut microbiota (primary outcome) was analyzed by Illumina sequencing. A 3-week supplementation with CG is well tolerated in healthy humans. CG induces specific changes in the gut microbiota composition, with *Eubacterium, Dorea* and *Roseburia* genera showing the strongest regulation. In addition, CG increased bacterial metabolites in feces including butyric, iso-valeric, caproic and vaccenic acids. No major changes were observed for the fecal BA profile following CG intervention. In summary, our work reveals new potential bacterial genera and gut microbiota-derived metabolites characterizing the interaction between an insoluble dietary fiber -CG- and the gut microbiota.

## Graphical abstract

**Figure:**
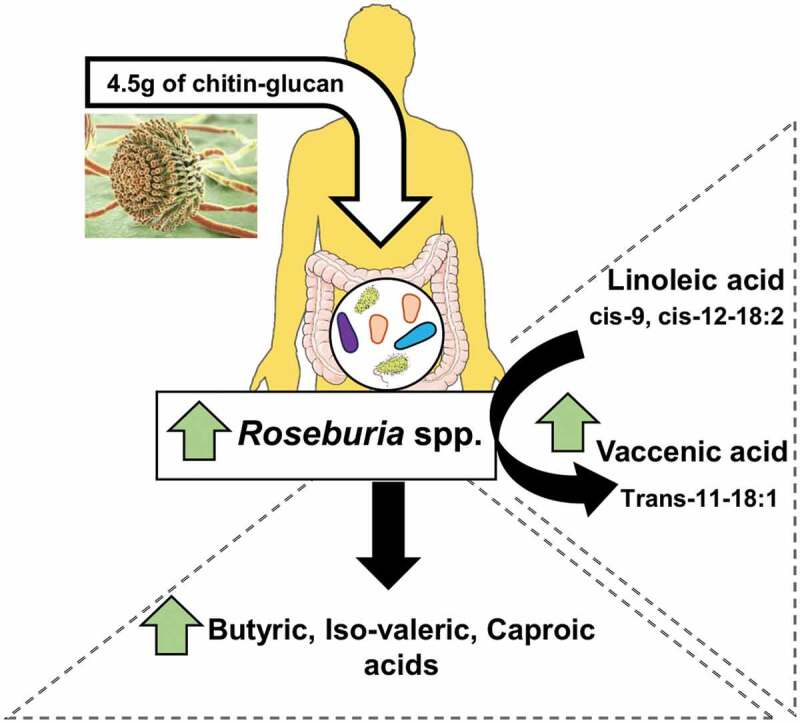

## Introduction

The nutritional interest of dietary fibers (DF) comes from the recognition of their benefits for health based on a large body of literature. DF is a category of non-digestible food components that includes lignin, cellulose, resistant starches, non-starch-polysaccharides, oligosaccharides, and analogous polysaccharides with associated health benefits.^1^ The concept of prebiotics, initially elaborated with non-digestible oligosaccharides specifically fermented by gut bacteria,^2^ was recently revisited.^3,4^ Prebiotics include fermentable DF which, through their interaction with the gut microbiota, modulate its composition and functions with beneficial effects for the host. On the basis of studies in animals and humans, it has been proposed that fermentable-prebiotic DF might increase satiety, improve metabolic disorders, and modulate gut-related immunity through mechanisms related to SCFA influencing endocrine and metabolic functions and intestinal epithelial integrity.^5–9^ However, it remains unclear which are the key health physiological effects generated by insoluble DF, and whether they rely on the gut microbiota.

In this context, chitin-glucan (CG) is a novel insoluble DF considered as safe food ingredient by the European Food Safety Authority.^10^ It is extracted from the cell walls of the fungi *Aspergillus niger*. For almost all fungi, the central core of the cell wall is a branched β-1,3/1,6 glucan that is linked to chitin via a β-1,4 linkage. Given CG insolubility, it is expected to be hardly fermented by the resident microbiota. However, using an *in vitro* approach with a Simulator of the Human Intestinal Microbial Ecosystem (SHIME), Marzorati et al. discovered that CG was fermented in all colon segments.^11^ This was shown by an increased distal colonic acidification, an enhanced SCFA production (mainly propionate and butyrate) and low levels of gas production. Our laboratory previously showed that a 4-week administration of CG significantly improved metabolic parameters in mice on a high fat (HF) diet (body weight gain, glucose tolerance, hepatic steatosis).^12^ In parallel to the metabolic outcomes, an increase of *Roseburia spp*., a butyrate-producing bacterium, was also detected in mice supplemented with CG.^12^ Another *in vivo* study demonstrated the beneficial effects of CG in lowering plasma triglycerides and reducing the area of aortic fatty streak deposition in hamsters fed with an atherogenic diet.^13^ In humans, in a 6-week, randomized, double-blind and placebo-controlled study, CG also exhibited beneficial effects by reducing blood levels of oxidized low-density lipoproteins (LDL).^14^ However, to our knowledge, no studies evaluated the impact of CG on both gut microbiota composition and on gut-derived metabolites such as the SCFA production in humans.

In the context of the project FiberTAG (Joint Programming Initiative “A Healthy Diet for a Healthy Life” 2017–2020 https://www.fibertag.eu/), we aimed at establishing a set of biomarkers of gut barrier function and bacterial co-metabolites linking DF intake and gut-microbiota related health effect.^15^ For this purpose, a monocentric longitudinal intervention study was conducted to characterize gut microbiota composition and major lipid and fiber-derived metabolites before and after a daily supplementation of 4.5 g of CG for 3 weeks in healthy volunteers.

## Results

### Subjects

**Figure 1.**
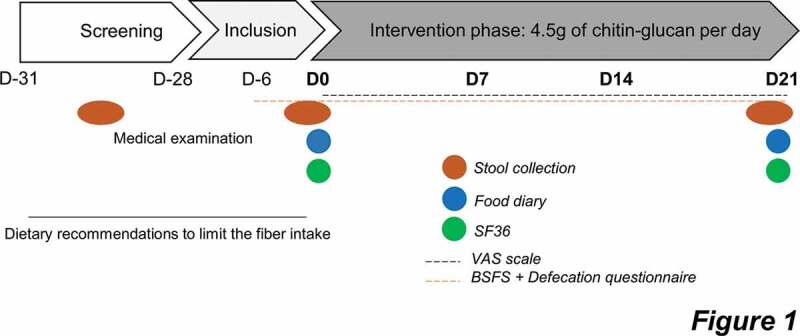
Protocol design of the intervention.

Sixteen subjects were initially included in the study. Fifteen subjects (seven men and eight women) completed the study; only one volunteer dropped out on the last test day (no reason was given). The mean age of participants was 21 years old (21 ± 1) and their body mass index was 22.1 ± 0.5 kg/m^2^. All volunteers (n = 15) were included in the analysis. The study was conducted from April to May 2018. The experimental design is presented in Figure 1. There was 96.5% compliance in the participants. Energy intake decreased by approximately 15% after 3 weeks of CG supplementation (Table 1). Macronutrient, alcohol, and fiber intake were not significantly affected by the intervention.

**Table 1.** Nutrient intake before and after 3 weeks of chitin-glucan supplementation in healthy volunteers.

(per day)	Baseline	3 weeks	p-value
Energy intake, kcal	1690 ± 114.60	1444 ± 75.07	.035*
Carbohydrates, g	193.9 ± 22.23	159.6 ± 10.26	.229
Lipids, g	64.52 ± 5.79	58.77 ± 4.01	.169
Proteins, g	63.51 ± 3.01	60 ± 4.10	.213
Alcohol, g	7.43 ± 3.2	1.67 ± 0.73	.082
TDF, g	14.35 ± 0.46	13.53 ± 0.85	.389
SDF, g	4.48 ± 0.22	4.59 ± 0.36	.454
IDF, g	9.03 ± 0.42	8.3 ± 0.61	.389

TDF: Total dietary fibers; SDF: soluble dietary fibers; IDF: insoluble dietary fibers. Fibers intake did not take into consideration the CG intake during the intervention. Data are expressed as mean ± SEM. *Mean values are significantly different from baseline (Wilcoxon matched-pairs test; *p* < 0.05).

One month before the start of the intervention, subjects were prescreened and followed a medical examination. Protocol starts at day 0, healthy subjects were asked to daily consume 4.5 g of CG during three weeks. The protocol ends at day 21. Test days took place at both day 0 (D0) and day 21 (D21) during that stool samples were collected. Patients were asked to fill out a food diary and questionnaires about gastrointestinal symptoms and quality of life (SF36: 36-item short form survey; VAS; 100-mm visual analog scales; BSFS: Bristol Stool Form Scale).

### Quality of life and gastrointestinal tolerance

The 36-item short form survey used allowed us to evaluate the level of eight health outcomes including components of both physical and mental health. In our study, CG supplementation did neither alter physical nor mental health of participants (Supplemental Figure 1). Those results suggested that 3 weeks of CG supplementation had no impact on the quality of life of human volunteers.

**Figure 2.**
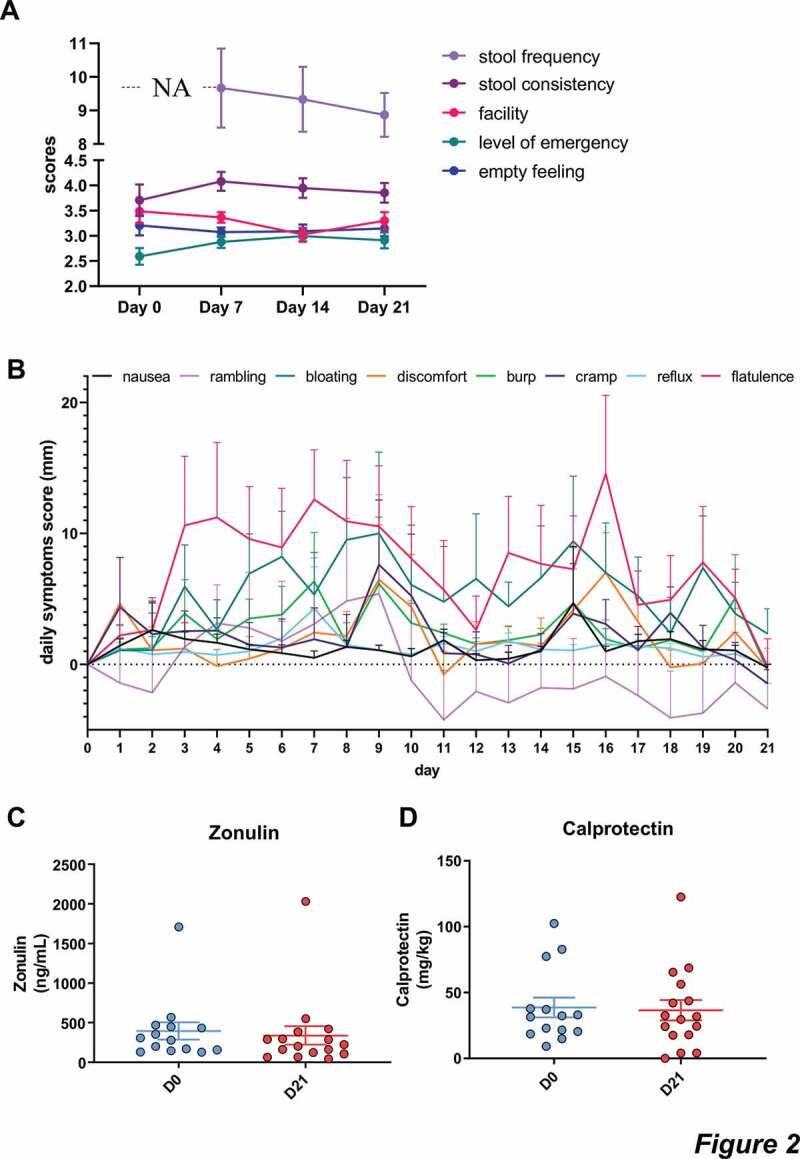
Gastrointestinal tolerance.

Throughout the intervention, no statistical differences were found regarding stool frequency (*p = .729*) or consistency (*p = .094*) and empty feeling (*p = .860*, Figure 2a). However, we observed a significant regulation of both defecation facility (*p = .048*) and the level of emergency (*p = .023*) by the treatment throughout the intervention, as assessed by mixed-effect model. Multiple comparisons only highlighted a significant change only between the day 14 and baseline, for both parameters (*p = .035* for defecation facility and *p = .020* for level of emergency, Figure 2a). In order to evaluate the tolerance to CG by the participants, VAS scores for eight gastrointestinal symptoms were reported every day during the intervention protocol at day 0 and day 21. The evolution of the gastrointestinal parameters between the baseline and the end of intervention were not significantly modified following the intake of fibers (Figure 2b).

**a**: results of the Bristol stool scale (stool frequency and consistency) and the defecation questionnaire measuring urgency, facility and emptying. Data are expressed as mean±sem. NA: non applicable. Mixed-effects analysis were performed for detecting the treatment effect throughout the intervention. Mixed-effects analysis showed a significant effect of treatment on the evolution of level of emergency, and defecation facility, during the intervention (level of emergency, *p* < .05). **b**: gastrointestinal symptoms including discomfort, nausea, flatulence, cramp, burp, bloating, rumbling and reflux. Mixed-effects analysis were performed for detecting the treatment effect throughout the intervention. No significant difference was observed for any symptoms. **c-d**: Fecal concentrations of zonulin and calprotectin.

We then analyzed the impact of CG on the gut barrier by measuring the levels of zonulin in the feces, known to regulate the tight junctions and for which a high concentration of this marker is associated with increased permeability.^16^ We did not observe any significant variation of fecal zonulin (*p = .119*, Figure 2c). In addition, we evaluated the concentration of fecal calprotectin, a marker for gut inflammation and we did not observe any changes for this marker after CG intervention (*p = .855*, Figure 2d).

### CG impact on gut microbiota composition

**Figure 3.**
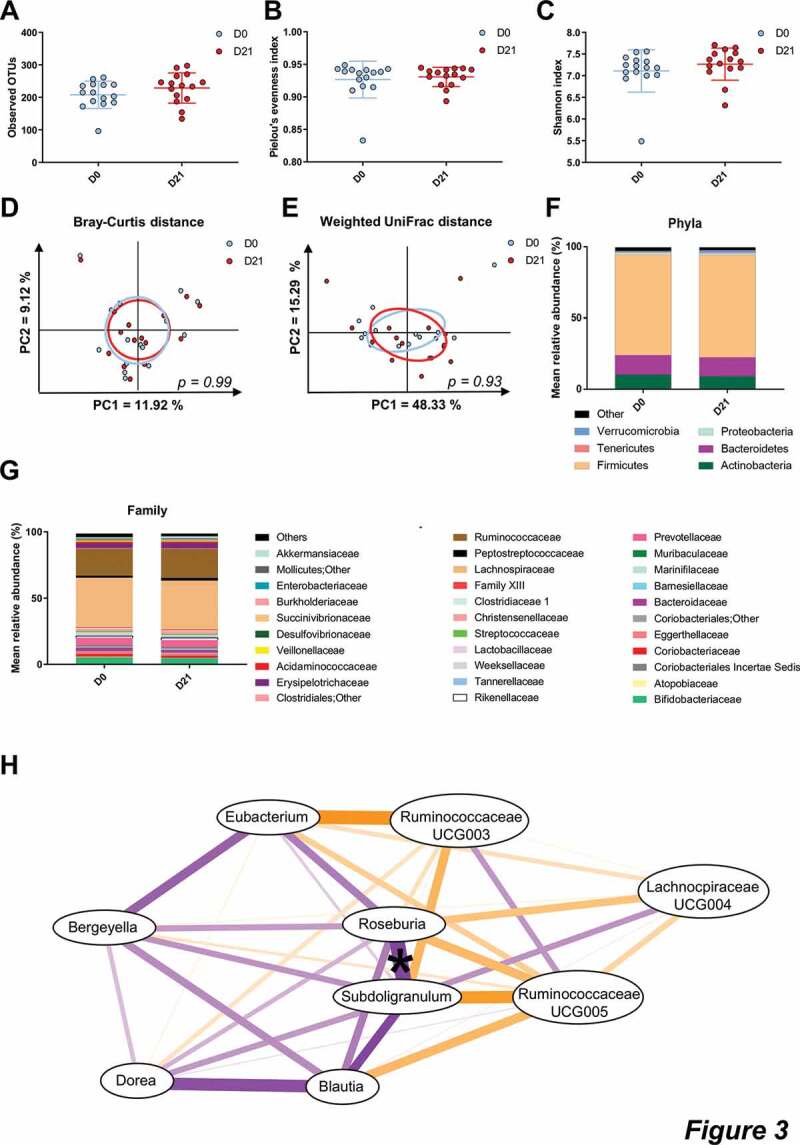
CG did not change the overall composition of the gut microbiota.

The impact of CG intake on gut microbial changes was analyzed by comparing the gut microbiota of participants at baseline and after 3 weeks of intervention. The α-diversity indices, related to bacterial richness (Observed OTU), evenness (Pielou) or both (Shannon), were not significantly affected by CG supplementation (Figure 3a-c). Moreover, the β-diversity characterizing the overall gut microbiota composition was not modified by the treatment as shown by the principal coordinate analysis of the Bray-curtis and the Weighted UniFrac distance (Figure 3d,e). No changes in the gut microbiota composition were observed after the CG intervention at the phylum level (figure 3f). At the family level, the only significant change detected was a decrease in the *Weeksellaceae* family (Figure 3g, Table 2).

**Table 2.** Bacterial taxa and ASV significantly different after 3 weeks of CG intake.

Taxa or ASV	Baseline	3 weeks	p-value < 0.5	q-value < 0.1
**Family**				
Weeksellaceae	1.196 ± 0.372	0.667 ± 0.238	.*023*	
**Genus**				
*Bergeyella*	1.196 ± 0.372	0.665 ± 0.237	.*018*	
*Blautia*	5.956 ± 0.563	4.957 ± 0.523	.*041*	
*Dorea*	2.244 ± 0.126	1.677 ± 0.166	.*004*	*0.049*
*Lachnospiraceae UCG-004*	0.087 ± 0.028	0.221 ± 0.06	.*025*	
*Roseburia*	1.473 ± 0.276	2.301 ± 0.405	.*008*	*0.087*
*Eubacterium*	4.588 ± 0.311	6.665 ± 0.	*<.001*	*0.008*
*Ruminococcaceae UCG-003*	0.218 ± 0.07	0.357 ± 0.085	.*029*	
*Ruminococcaceae UCG-005*	0.681 ± 0.148	1.31 ± 0.249	.*025*	
*Subdoligranulum*	1.74 ± 0.226	1.312 ± 0.182	.*028*	
**ASV**				
*ASV12_Collinsella sp.*	1.501 ± 0.296	1.002 ± 0.209	.*036*	
*ASV65_Blautia sp.*	0.74 ± 0.091	0.4 ± 0.071	.*001*	
*ASV70_Anaerobutyricum [Eubacterium] hallii (99%)*	0.358 ± 0.133	0.246 ± 0.098	.*036*	
*ASV76_Coprococcus comes (99%)*	0.548 ± 0.13	0.297 ± 0.086	.*011*	
*ASV81_Subdoligranulum sp.*	0.352 ± 0.109	0.131 ± 0.066	.*014*	
*ASV107_Eubacterium sp.*	0.331 ± 0.118	0.176 ± 0.08	.*036*	
*ASV116_Anaerobutyricum [Eubacterium] hallii (99%)*	0.346 ± 0.131	0.159 ± 0.092	.*036*	
*ASV130_Terrisporobacter sp.*	0.171 ± 0.058	0.445 ± 0.113	.*037*	
*ASV155_Blautia sp.*	0.593 ± 0.069	0.363 ± 0.077	.*017*	
*ASV156_Blautia sp.*	0.34 ± 0.126	0.204 ± 0.08	.*035*	
*ASV173_Unclassified Ruminococcaceae*	0.263 ± 0.097	0.131 ± 0.051	.*036*	
*ASV241_Roseburia hominis (99%)*	0 ± 0	0.274 ± 0.089	.*022*	
*ASV730_Actinomyces sp.*	0.145 ± 0.039	0.049 ± 0.023	.*021*	

Data are expressed as mean percentage of relative abundance and presented as mean ± SEM. Wilcoxon matched-pairs test, significant if *p* < 0.05 (FDR correction; q < 0.05). For ASV identification, species name is indicated when the identity is > 98%.

**a-c**: Measures of alpha-diversity: Observed OTUs, Pielou’s evenness measure and Shannon. Data are expressed as mean±SEM. Wilcoxon matched-pairs test between D0 and D21. **d-e**: Principal coordinates analysis (PCoA) of the β-diversity indexes Bray-Curtis and Weighted UniFrac. *p*-values refer to Monte Carlo rank test performed on R software. **f-g**: Barplots of relative abundance of phylum and family levels accounting for more than 1% and 0.1% respectively. **h**: Correlation network analysis of genus significantly changed by CG. Spearman correlation, *p < .05. Orange lines indicate negative correlations, purple lines represent positive correlations.

Using univariate analyzes, we found that 3 weeks of CG intake significantly altered relative abundance of nine bacterial genera (Table 2), increasing *Lachnospiraceae UCG-004, Roseburia, Eubacterium, Ruminococcaceae UCG-003* and *UCG-005*, whereas it decreased *Subdoligranulum, Bergeyella, Blautia* and *Dorea*. The variation of *Subdoligranulum* genus was significantly correlated with the changes of *Roseburia* spp. (Figure 3h). In order to see if the level of these nine genera could be influenced at the individual level independently of CG supplementation, we compared their relative abundance at two time points before CG intervention: at time −30 days and at time 0 (just before the intervention). As shown in Supplemental Table 1, most of these genera were stable prior to CG intervention (no difference at −30 days versus time 0). Only the relative abundance of *Bergeyella* and *Dorea* was less expressed 30 days before the study than at day 0.

Since the abundance of specific amplicon sequence variants (ASV) within the same genus could be differentially affected by the treatment, we also performed the gut microbiota analysis based on ASV variation (Table 2). We found only two ASV significantly up-regulated by CG intervention: ASV241 related to the species *Roseburia hominis*, consistently with the increase of *Roseburia* genus, and ASV130 belonging to *Terrisporobacter* genus, which was not impacted by CG. Another set of 11 ASV was decreased by 3-weeks of CG intake, including ASV belonging to *Blautia, Coprococcus, Subdoligranulum, Actinomyces, Collinsella* or *Eubacterium*. Some of these genera (*Blautia, Eubacterium and Subdoligranulum*) were also impacted by CG.

These data suggest that a 3-week CG supplementation did not induce major changes in the overall gut microbiota composition but resulted in rather specific changes to bacterial genera and species.

### CG impact on fecal microbial metabolites

**Figure 4.**
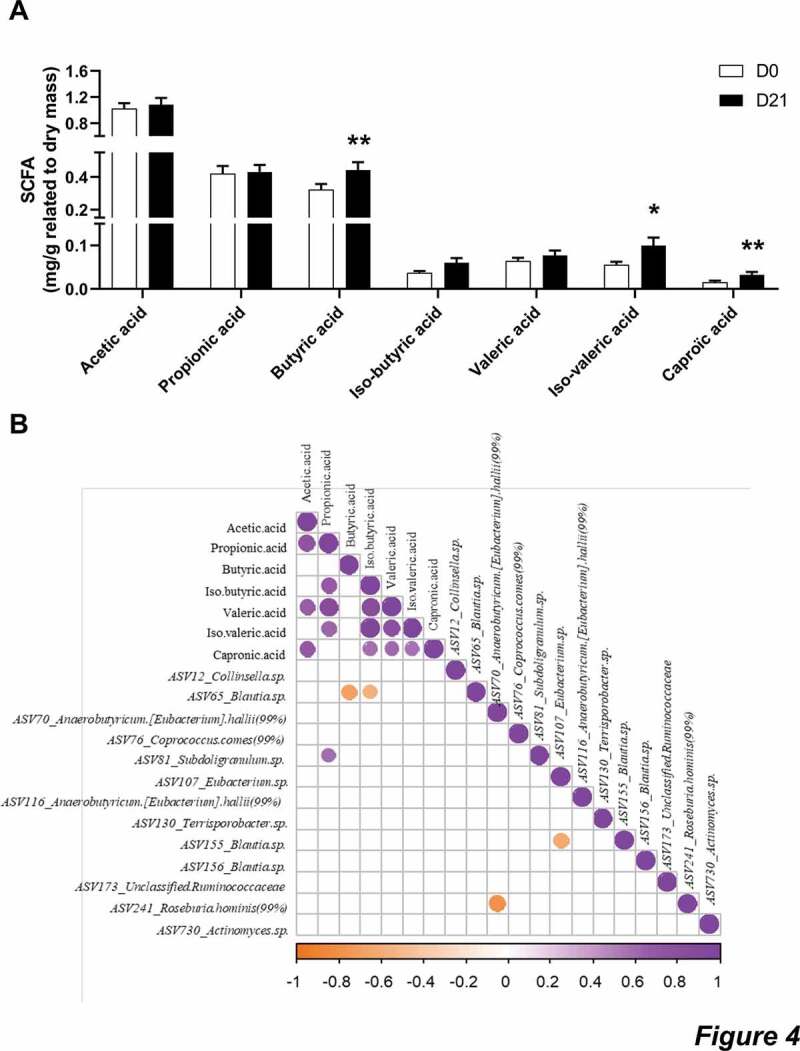
CG increased the fecal concentration of butyric, iso-valeric and caproic acids.

We measured the fecal concentrations of SCFA (Figure 4a). CG intake led to an increase of butyric, iso-valeric and caproic acids (*p = .004, p = .049* and *p = .003*, respectively) and tended to increase iso-butyric acid (*p = .091*). Fecal acetic, propionic and valeric acids remained unchanged after 3 weeks of CG intake. Correlative analysis between the measured SCFA and the ASV regulated by CG showed that the decrease of the ASV65 belonging to *Blautia* is correlated with the increase of both butyric and iso-butyric acids (*r = −0.688* and *p = .008* for butyric acids; *r = −0.574* and *p = .03* for iso-butyric acids, Figure 4b).

**a**: Fecal concentrations of SCFA. Data are expressed as mean ± sem. Wilcoxon matched-pairs test between D0 and D21. *p < .05 and **p < .01. B: Heatmap of Spearman’s correlations between the ASV significantly modified by CG treatment and the fecal concentrations of SCFA. The presence of a circle indicates that the correlation is significant, *p* < .05.

**Figure 5.**
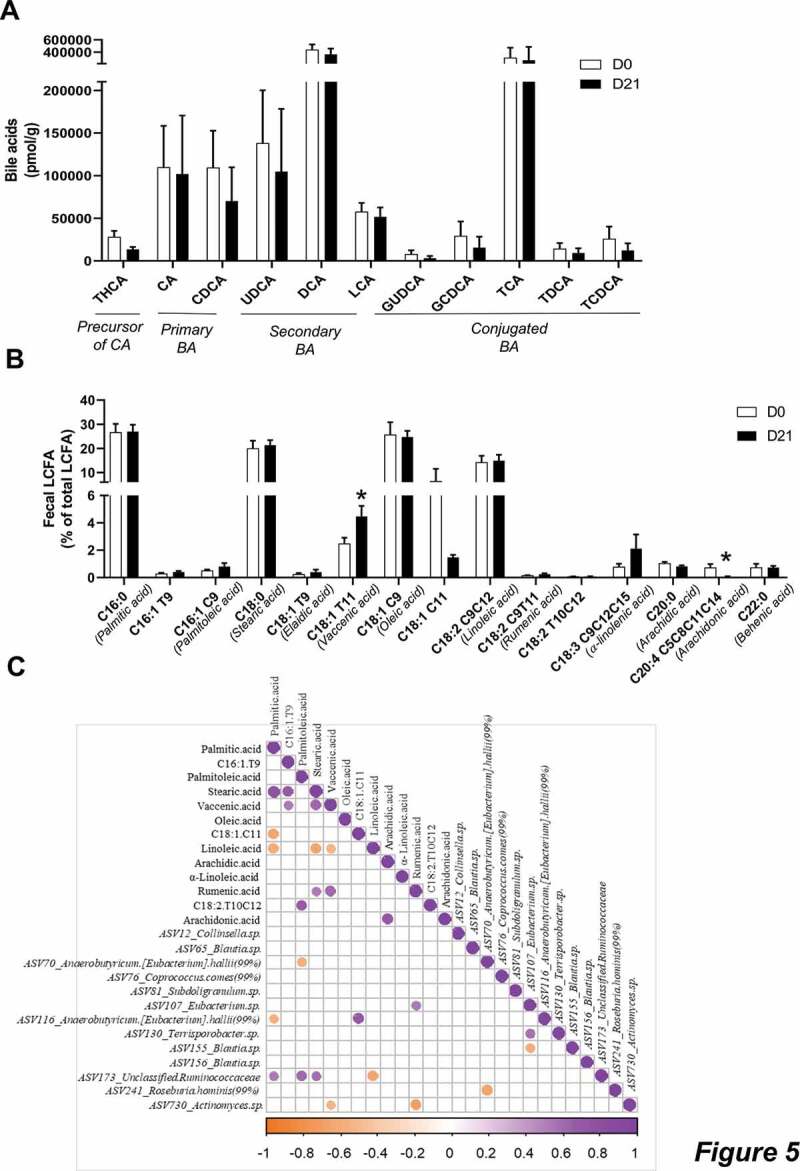
CG increased the fecal concentrations of vaccenic acid.

We found minor differences in the fecal BA concentrations after the intervention. The only detectable effect was a tendency to decrease trihydroxycholestanoic acid (THCA), a precursor of cholic acid synthesis, whose change approached significance (*p = .09*, Figure 5a).

**a**: Data are normalized to the mass of dry matter and are expressed as mean ± sem of the ratio between the signal of the analyte and the signal of its internal standard. **b**: Percentage of LCFA/total LCFA detected in the feces of human volunteers. Data are expressed as mean ± sem. Wilcoxon matched-pairs test between D0 and D21. *p < .05. **c**: Heatmap of Spearman’s correlations between the ASV significantly modified by CG treatment and the fecal concentrations of LCFA. The presence of circle means that the correlation is significant, *p* < .05.

Finally, we also measured fecal LCFA, including conjugated-polyunsaturated fatty acids (cPUFA) or trans-fatty acids (Figure 5b). We found that CG supplementation significantly increased the levels of vaccenic acid (C18:1 *trans*-11) whereas it decreased the levels of arachidonic acid (C20:4 *cis*-5, *cis*-8, *cis*-11, *cis*-14). We found that the regulation of vaccenic acid correlated with the decreased ASV730 belonging to *Actinomyces*, upon CG intervention (*r = −0.538* and *p = .038*, Figure 5c).

## Discussion

In the current study, we have shown that 3 weeks of CG intake was well tolerated by healthy subjects, had no effect on gut transit or well-being, and induced significant changes in the gut microbiota composition. In addition, CG intake also led to a significant regulation of some fecal gut microbiota-derived metabolites that correlated with specific bacteria modulated by CG.

CG was previously administered to healthy volunteers in order to evaluate its effects on oxidized low-density proteins.^14^ In their study, the authors did not report any adverse effects after six weeks of CG supplementation (with 1.5 g or 4.5 g/day of CG). Coherently, we noticed that 3 weeks of CG intake neither altered the stool frequency nor consistency. However, the treatment significantly increased the level of emergency and decreased defecation facility throughout the study to finally become similar, compared to day 0, at the end of the study. Although this change in bowel habits observed at the day 14 compared to baseline are not favorable, it is important to note that these effects are disappearing further on upon treatment. In addition, the CG supplementation did not induce gastrointestinal symptoms in the participants. Moreover, CG did not alter the general health, assessed by questionnaires and the stool form score assessed by the BSFS was around 4, corresponding to a normal transit, all along the study.^17^ The intake of fibers can be associated with side-effects such as gastrointestinal symptoms,^18^ partly due to a rapid fermentation causing gas production or bloating. It is interesting to remind that CG is slowly and gradually fermented as demonstrated *in vitro*,^11^ and did not induce gastrointestinal symptoms in our study. Moreover, side effects could lead to poor compliance and intake of less than the recommended dose, that it was not the case during the intervention with an observed 96% compliance. This confirms that CG seems to be well-tolerated by healthy volunteers.

We then assessed the concentrations of fecal zonulin and fecal calprotectin, which are biomarkers of gut barrier and gut inflammatory disorders, respectively.^19,20^ The fecal level of these two markers was not affected by CG treatment.

Importantly, our intervention study allowed to evaluate, for the first time in humans, the bacterial changes upon CG supplementation. Previous studies highlighted that fungal CG intake was associated with an increase of the butyrate-producing bacteria *Roseburia* spp. in both an *in vitro* model using a SHIME and in a preclinical model of high-fat diet mice.^11,12^ Here, we confirmed in healthy human volunteers that 3 weeks of CG supplementation is sufficient to increase the relative abundance of the genus *Roseburia* spp. and particularly an ASV belonging to the *Roseburia hominis* species. Given that CG is consistently linked with an increase in *Roseburia* spp. (i.e. *in vitro, in vivo* in mice, and in healthy humans) led us to propose *Roseburia* spp. as a target of CG intake. Furthermore, *Roseburia* strains are known for their considerable ability to utilize diet-derived polysaccharides and in turn produce butyrate.^21^ In our study, we highlighted a consistent increase of butyric acid in fecal samples after CG supplementation. In association with *Roseburia* genus, literature indicated that another abundant group of butyrate-producing bacteria in the human colon is *Faecalibacterium prausnitzii*.^21^ In accordance with these findings, we only observed that an ASV belonging to *Faecalibacterium* genus tended to increase upon CG intervention (*p = .097*, data not shown).

Other species regulated by CG in our study are also known for their ability to produce butyrate, including *Anaerostipes hadrus* or *Anaerobutyricum* [*Eubacterium*] *hallii*.^22^ However, we found that CG significantly decreased the abundance of two ASV belonging to *Anaerobutyricum* [*Eubacterium*] *hallii* and only tended to increase the abundance of an ASV belonging to the *Anaerostipes* genus (*p = .1*, data not shown). CG also strongly increased *Eubacterium* genus. Interestingly, this genus was positively correlated with some circulating cPUFA (*cis*-9, *cis*-11-18:2; *trans*-9,*trans*-11-18:2 and *cis*-9,*trans*-11,*cis*-15-18:3) in a cohort of obese women supplemented with inulin-type fructans as prebiotics.^23^ In our study these specific cPUFA were not detected in the feces of healthy subjects. However, it may be unlikely that similar associations occurred between bacterial genera and cPUFA detected in different samples such as feces or in plasma. We also found that CG intake increased the fecal levels of iso-valeric acid, a product from proteolytic fermentation, especially issued from leucine bacterial catabolism.^24^ Furthermore, we detected an increase of fecal caproic acid. This metabolite has been recently shown to be produced from lactate oxidation.^25^ In this model, lactate oxidation provides acetyl-CoA which enters elongation process to form butyrylCoA and hexanoylCoA (also called caproylCoA). Regarding this hypothesis, the increase of both butyric and caproic acids following the CG intake could be consistent in our study.

Secondary bile acids are produced by gut microbes via biotransformation of host-derived primary bile acids.^26^ Regarding the bacterial metabolites produced from cholesterol, we did not detect any differences in bile acid profile after CG intake, except a tendency of CG to decrease a precursor of bile acid synthesis, the THCA. Secondary bile acids are not the sole lipidic metabolites produced by the gut microbes from lipids. Indeed, PUFA may be also reduced by bacteria, leading to trans and conjugated fatty acids. We observed that CG intake significantly increased the concentration of fecal vaccenic acid, another type of lipid-derived bacterial metabolites.^27^ It is interesting to observe an increase of vaccenic acid in parallel to an increase of *Roseburia* after the CG supplementation. Indeed, a previous study identified the ability of *Roseburia* spp. to actively metabolize linoleic acid, forming either vaccenic acid or an hydroxy-18:1 fatty acid.^28^ Interestingly, two species are able to produce vaccenic acid: *Roseburia hominis* and *Roseburia inulinivorans*. The ability of these species to produce vaccenic acid was confirmed in pure cultures from linoleic acid in deuterium-oxide enriched medium.^29^ Although we did not find any statistical significant correlation between vaccenic acid and an ASV belonging to *Roseburia hominis*, a consistent increase of this metabolite in parallel to a higher abundance of this ASV was observed in participants receiving CG, suggesting that the increase of vaccenic acid could also be a biomarker of CG interaction with gut microbiota. Interestingly, vaccenic acid (C18:1 *trans*-11) level correlated positively with other trans isomers of mono-unsaturated fatty acids (C16:1 *trans*-9), and with the final product of bacterial reduction of rumenic acid, stearic acid (C18:0); in addition, it inversely correlated with linoleic acid (C18:2 *cis*-9, *cis*-12), the precursor of rumenic acid reduction pathway of bacteria. This is in favor of the bacterial origin of vaccenic acid increase by CG. Several studies evaluated the impact of vaccenic acid on health, and suggest a beneficial effect by this LCFA on cardiometabolic disease risk, linked namely to changes in blood lipids, both in animal models and in humans.^30–34^ The contribution of dietary supplementation versus the “endogenous”, meaning gut microbial production of vaccenic acid to lipid metabolism, remains to be established.

In the present study, the dose of CG administered corresponds to the recommendations made by EFSA (range from 2 to 5 g per day).^10^ Regarding some other commonly used prebiotics, a similar dose of arabino-xylan-oligosaccharides (AXOS, 4.8 g/day during 3 weeks) was sufficient to induce a selective increase of *Bifidobacterium* in healthy individuals.^35^ In another work, 10 g of wheat bran extract (enriched in AXOS) consumption for 3 weeks increased *Bifidobacterium* in healthy adults and increased butyric acid (*p = .05*) by approximately 4.5% compared to a previous placebo period.^36^ Here, we observed a 28% increase in fecal butyric acid after CG intervention, compared to baseline. For other prebiotics, including inulin-type fructans, a higher dose is commonly used (around 15–20 g/day) to induce significant changes on the gut microbiota, with mostly a focus on the bifidogenic effect.^5,37^ A dietary intervention with a higher consumption of vegetables enriched in fructans allowed to largely increase the intake of fructans (that reached 15 g per day), and lead to the increase in bifidobacteria.^38^ Three weeks of daily supplementation (16 g/day) with a mix of inulin and fructo-oligosaccharides (FOS) in healthy individuals did not change SCFA, increased *Bifidobacterium* and decreased *Dorea, Coprococcus* and *Ruminococcus*.^39^ Interestingly *Dorea* genus and an ASV belonging to *Coprococcus* also decreased in our study with CG. Another study from *Baxter et* al. revealed that not all fermentable fibers are equally capable of stimulating SCFA production.^40^ Contrary to a high-amylose maize seeds (20–24 g/day) or inulin from chicory root (20 g/day), resistant potato starch (28–34 g/day) significantly induces a 29% increase of fecal butyric acid in healthy participants. Given the ability to increase fecal butyric acid after CG supplementation and knowing the beneficial health effect attributed to butyrate, it could be relevant to test this fiber in the context of metabolic and bowel disorders.^41^

One limitation of our study is the lack of a control group (placebo intervention) since we made the choice to compare the data after versus prior intervention in the same subjects. A previous study supported the strategy to use the same individual as own control in order to highlight microbial signature of a prebiotic DF.^38^ By taking advantage of samples collected in 13 individuals enrolled in the chitin-glucan intervention protocol 30 days before the start of the study, we were able to show that a shift in the gut microbiota composition after 3 weeks – including in particular an increase of *Roseburia* and *Eubacterium* genera- could clearly be attributed to CG treatment, and is not due to intra-individual variations of microbiome with time.

In conclusion, our study performed in healthy volunteers identified some fecal biomarkers of CG interactions with the gut microbiota. Previous evidence obtained in preclinical models suggest that CG intake could offer many benefits in the context of metabolic alterations. In this study, we confirm that some CG effects could be interesting to investigate in the context of metabolic disorders. This is for instance the case for the increase of *Roseburia* spp., as well as for the increase in butyric acid and vaccenic acid levels. In consequence, we think that our future effort will focus on the interest of CG supplementation in the management of metabolic alterations patients with cardiometabolic disorders, a study under investigation in the FiberTAG project.^15^

## Materials and methods

### Power analysis and sample size

The primary outcome of the study was the gut microbiota composition by Illumina sequencing of 16S rRNA. Sample size was estimated using the software PASS 14.0.7 and the paired mean power analysis test. Since it is an exploratory experiment aiming to study for the first time the impact of CG on the gut microbiota in humans, we calculated the sample size based on a study performed with prebiotic with similar outcomes^38^ and by hypothesizing a regulation of an abundant genus previously regulated *in vivo* by CG, namely *Roseburia*. In order to detect a significance difference for an abundant genus, we calculated sample size for a difference of 2% in the relative abundance with a variance of 2% between and within individuals, a significance of 0.05 and a power of 80%. For this, 15 subjects were sufficient and 16 subjects were included in the study to allow for a 10% drop out.

### Human study

The recruitment was conducted from February to March 2018. Thirty-four healthy males and females were recruited by the Center of Investigation in Clinical Nutrition (CICN), the platform from the Université catholique de Louvain (Louvain-la-Neuve, Belgium) and underwent the screening test. Among them, 16 subjects were included in the study based on the predefined inclusion and exclusion criteria and therefore received the allocated intervention. Fifteen completed the study.

For this study, healthy subjects were chosen to investigate the relevance of supplementing CG in the context of a healthy lifestyle. The inclusion criteria were: men or women, aged 18–40 years, body mass index (BMI) between 18 and 25 kg/m^2^, Caucasian, nonsmoker, in good general health as evidenced by medical history and physical examination, the use of effective contraception for women, and need to be H_2_-producer. Subjects were pre-screened by a phone or mail questionnaire and they were then invited to perform a lactulose screening test to assess if they are H_2_-producers and stool were collected. Screening lactulose test was performed at least 4 weeks before the intervention in order to assess if they are producer of H_2._ Briefly, fasted subjects (at least 10 hours) received on oral load of 10 g of lactulose; then, exhaled H_2_ were measured every 30 minutes during 4 hours (using Lactotest 202, Medical Electronic Construction, MEC). A minimal increase of 10 ppm of H_2_ during three successive measurements is needed to select the H_2_-producers.^42^ No increase should be observed in the first 30 minutes to avoid subjects with small intestinal bacteria overgrowth. Participants provided informed consent as well as stated willingness to comply with all study procedures and availability for the duration of the study.

The exclusion criteria were: gastro-intestinal disorders, chronic or intestinal diseases (such as ulcers, diverticulitis or inflammatory bowel diseases), smoking, current or recent use of antibiotics, pro/prebiotics (as a dietary supplement) or any products affecting the gut transit (such as laxatives) within 4 weeks before starting the study, use of drugs that modify the composition of gut microbiota (antidiabetic drugs, cholesterol-lowering drugs, and proton pump inhibitors), pregnant or lactating woman (and woman who did not use highly effective contraception), psychiatric problems (and/or using antipsychotics), chronic intake of drugs, following a particular diet (e.g., vegetarian, high-fiber, or high-protein diets), allergy or food intolerance (e.g., lactose or gluten), presence of allergy or intolerance to one component of the tested product, excessive alcohol consumption (more than 3 units/day) and participation in another clinical trial 1 month before the screening visit.

Following the screening test, selected subjects performed the medical examination with the physician investigator to verify that they were healthy. After this medical examination, subjects were advised by a dietician to avoid diets too rich in DF, from at least two weeks before the intervention. Food products consumption containing high amounts of dietary fibers (including whole grains, artichokes, Jerusalem artichokes, salsifies, leeks, onions) was limited to once a week. During the intervention, subjects were contacted frequently by mail or phone to verify that they still met the inclusion/exclusion criteria, that they followed the dietary advices and for the stool collection. Moreover, subjects were asked to complete an electronic questionnaire (via a software called REDCap, Research Electronic Data Capture) every day during the intervention, which allowed monitoring subjects’ side effects and compliance.

This study was approved by the local ethical committee (Comité d’Ethique Hospitalo-Facultaire UCLouvain/Cliniques Universitaires Saint-Luc) and written informed consent was obtained from all subjects before inclusion in the study. The trial was carried out in accordance with the Good Clinical Practice (GCP) as required by the following regulations: the Belgian law of 7 May 2004 regarding experiments on the human persons and the EU Directive 2001/20/EC on Clinical Trials. The trial was registered at ClinicalTrials.gov under identification number NCT03505177; Registered 23 April 2018).

#### Study design

This is a monocentric, longitudinal interventional study consisting of a daily supplementation of 4.5 g of CG during three weeks. Empty and unused packets were returned to measure compliance. The detailed protocol is schematized in Figure 1. Within 2 days before day 0 and day 21, all subjects were asked to provide fresh stool samples. The detailed protocol is schematized in Figure 1. The volunteers were their own control in the design of the study. The subjects were asked to complete a food diary for 3 days at day 0 and day 21 to assess the impact of the nutritional intervention on nutrient intake. The Nubel Pro program and the table of composition from Nubel 2010 were used to assess macronutrient and total fiber intake.

During the intervention, the participants were asked to fill out 100-mm visual analog scales (VAS) describing their gastrointestinal symptoms (discomfort, nausea, flatulence, cramp, burp, bloating, rumbling and reflux) every day. Each subject completed separate scales, one for each symptom. The scales were scored by measuring the distance (in millimeters) from 0. The stool frequency and consistency were investigated through daily self-assessment using the Bristol Stool Form Scale (BSFS), 3 days before the intervention and throughout the entire CG intervention. For stool frequency, scores recorded were added per week, meaning that the first result is presented seven days after the start of the study. The BSFS classifies stools into seven categories, including type 1, separate hard lumps, like nuts; type 2, sausage-shaped, but lumpy; type 3, like a sausage but with cracks on the surface; type 4, like a sausage or snake, smooth and soft; type 5, soft blobs with clear-cut edges; type 6, fluffy pieces with ragged edges, a mushy stool; type 7, watery, no solid pieces.^17,43^ A defecation questionnaire for the stools produced during the last 24 hours was also submitted and completed every day (from day −6 until day 21). A 5-point Likert’s scale assessed the ease of passage (from “very difficult” to “very easy”), the level of emergency (from “not at all urgent” to “very urgent”) and the empty feeling (“not at all empty” to “completely empty”). In addition, a generic “quality of life” questionnaire (a 36-item short form (SF36) survey) that has been widely validated,^44,45^ has been completed at day 0 and day 21. The items are grouped into eight domain scores: physical functioning, role limitations due to physical health, pain, general health, energy, social functioning, role limitations due to emotional problems and emotional well-being. Domain scores can be collapsed to a physical component summary “Physical health” (PCS) and a mental component summary “Mental health” (MHS). Each score ranges from 0 to 100, with higher values representing better self-perceived health-related quality of life.

#### Study outcomes

The primary outcome of the study was the evaluation of 3-weeks of CG supplementation on the gut microbiota composition. Secondary outcomes measures concern the analysis of fecal concentrations of gut microbiota-derived metabolites (including BA, LCFA and SCFA).

### Fecal sample collection and processing

Stool samples were collected (within 4 days before each test day: *n = 15* for D0 and *n = 15* for D21) in tubes labeled with each individual’s code number, frozen (and transported to the lab) at −20°C and then stored at −80◦C for the following analysis. During the screening test (30 days prior to intervention), stool samples were also collected in order to evaluate potential time-effect based on the 16S rRNA gene sequencing prior intervention (*n = 13*).

### Fecal microbiome sequencing and analysis

Bacterial DNA was extracted from fecal samples using the QIAamp DNA Stool Mini Kit (QIAGEN, Hilden, Germany), following the ‘Protocol Q’ that was described by Costea and colleagues,^46^ with a slight modification: a reduction in time of bead beating step. Cells are mechanically lysed by running the Fastprep™ Instrument for 2 min at max speed (beating 1 min and resting 5 min).

Library construction and the Illumina sequencing protocol has been previously described in detail.^47^ In brief, the V5-V6 regions of the 16S rRNA gene were targeted for PCR amplification using primers 784 F [5′-RGGATTAGATACCC-3′] and 1064 R [5′-CGACRRCCATGCANCACCT-3′]. 16S rRNA gene amplicons were sequenced by the MiSeq platform (300 bp paired-end length) at the University of Minnesota Genomics Center. All samples of this study were sequenced in the same run.

Paired-end reads were merged, demultiplexed and conducted quality control implementation (length = 281 bp, mean sequence quality score≥30) using QIIME2^48^ pipeline with DADA2,^49^ which we refer to herein as amplicon sequences variants (ASV). The average of the sequence in all samples (n = 31) was 29,373. An even depth of 18,750 sequences per sample was used to conduct microbiome diversity. We assigned the sequences to taxonomic categories including kingdom, phylum, class, order, family and genus levels using a pre-trained Naive Bayes classifier based on Silva 132 99% OTUs database.^50^ Phylogenetic position of ASV is presented in the supplemental Table 2 and is based on the Silva database using the Qiime2 pipeline. For significant ASV, in order to have a higher resolution of the ASV identification, online 16S rRNA databases on both NCBI blast and JGI IMG platforms were also used.

Raw sequences are deposited into the Sequence Read Archive (SRA) of NCBI (http://www.ncbi.nlm.nih.gov/sra) and can be assessed with the accession number PRJNA636138.

### SCFA analysis

For SCFA analysis the native fecal samples were homogenized and subsamples of 400–500 mg were diluted 1:4 in ultrapure water and stored at −80°C. Prior to analysis 0.1 ml of 50% ortho-phosphoric acid (AppliChem GmbH, Darmstadt, Germany) was added before the samples were filtered using polyester syringe filters (REF: 729033; Macherey-Nagel GmbH & Co. KG, Düren, Germany). Afterwards, 1 µl filtrate was analyzed using a capillary gas chromatograph (HP6890 Series; Hewlett Packard Corp., Paolo Alto, California, USA) with a flame ionization detector using the column OPTIMA-FFAP (REF: 726344.10; Macherey-Nagel GmbH & Co. KG, Düren, Germany) with standards for all SCFAs measured (all: Merck Schuchardt OHG, Hohenbrunn, Germany). Fecal dry mass was assessed by drying 300–500 mg of native sample overnight at 103°C.

### Markers of intestinal permeability

Zonulin and calprotectin were measured using enzyme-linked immunosorbent assay kits (K5600; K6927; Immundiagnostik AG, Bensheim, Germany) following the manufacturer’s protocol. The fecal samples were diluted to the working concentration in sample buffer using stool sample tubes (K6998SAS; Immundiagnostik AG, Bensheim, Germany).

### Bile acids analysis

Bile acids were analyzed using an LTQ-Orbitrap mass spectrometer (ThermoFisher Scientific) coupled to an Accela HPLC system (ThermoFisher Scientific) with a method adapted from *Guillemot-Legris et al*.^51^ Briefly, 5 mg of lyophilized feces were homogenized in ice-cold distilled water and proteins were precipitated using acetone containing seven deuterated bile acids used as internal standards. Samples were next centrifuged, and the supernatant was evaporated to dryness under nitrogen steam. The resulting residue was resuspended in methanol and injected in the HPLC-MS system.

### LCFA analysis

To determine the LCFA profile in feces, we used 40 mg of previously lyophilized feces during 48 h (Labconco, freeze dryer 4.5). Forty microliter of C19 were added as extraction standard for homogenization with methanol:chloroform (1:2 V/V) by brief sonication in ice (Labsonic U, B. Braun). Homogenates were then filtered with Whatman filters n°1 (10 μm of porosity). Filters were rinsed with 2 ml of chloroform and 1 ml of methanol. Homogenates were purified with KCl 0.88% and KCl 0.88%: methanol (1:1 V/V). After centrifugation (1500 g, 5 min), the chloroform phase was collected in new tubes and evaporated under nitrogen flux until samples were completely dry. The esterified fatty acids were then subjected to alkaline hydrolysis (saponification) and free fatty acids were methylated and quantified by gas chromatography with flame ionization detector as previously described.^27^

### Statistical analysis

Data are expressed as mean ± SEM. Mixed-effects models were used for analyzing the gastrointestinal tolerance of CG throughout the study and performed on JMP Pro 14 software (with time as fixed effects and subjects as random effects). For markers of intestinal permeability, gut microbiota composition and gut-derived metabolites, statistical analyses were evaluated using a Wilcoxon paired test (from baseline to 21 days of intervention). For gut microbiota analysis, relative abundances performed in Qiime2 are expressed as means and SEM and were calculated on R for each taxa and ASV. To avoid analyzing spurious sequences, ASV with a relative abundance below 0.1% in all samples were removed. The same cutoff was applied for analysis of bacterial genera. At the genus level, if there were multiple taxa groups that all had the same genus name and belonged to the same family, we combined them together. For instance, *Eubacterium genus* includes *[Eubacterium] brachy group, [Eubacterium] nodatum group, [Eubacterium] eligens group, [Eubacterium] fissicatena group, [Eubacterium] hallii group, [Eubacterium] ruminantium group, [Eubacterium] ventriosum group and [Eubacterium] xylanophilum group.*

The *p*-value of the Wilcoxon test was adjusted (q-value, significant if q < 0.05) to control for the false discovery rate (FDR < 0.05) for multiple tests according to the Benjamini-Hochberg procedure.^52^

Beta-diversity indices were evaluated on Qiime2 and visualized with a PcoA performed on R software, using ade4 package. A Monte Carlo rank test was assessed for Beta-diversity based PcoA.

Correlation network analysis was visualized using the qgraph package on R software.

Associations between the changes of ASV significantly regulated by CG and the changes of gut-derived metabolites between the day 21 and baseline were assessed by Spearman’s correlation tests. A significance level of *p* < .05 was adopted for all analyses. Heatmaps of correlation were visualized with the corrplot package on R software.

## Supplementary Material

Supplemental Material

## Figures and Tables

**Table:** 

BA	bile acid
CA	cholic acid
CDCA	chenodeoxycholic acid
CG	chitin-glucan
DCA	deoxycholic acid
DF	dietary fibers
FA	fatty acid
GCDCA	glycochenodeoxycholic acid
GUDCA	glycoursodeoxycholic acid
LCA	lithocholic acid
cPUFA	conjugated polyunsaturated fatty acid
SCFA	short-chain fatty acid
TCA	taurocholic acid
TCDCA	taurochenodeoxycholic acid
THCA	trihydroxycholestanoic acid
TDCA	taurodeoxycholic acid
UDCA	ursodeoxycholic acid
