# Supplementary material for: Metabolite profiling reveals the interaction of chitin-glucan with the gut microbiota
Source: Gut Microbes. 2020 Sep 6;12(1):1810530. doi: 10.1080/19490976.2020.1810530 (PMC7524357; doi:10.1080/19490976.2020.1810530)
Supplement: Supplemental Material [file KGMI_A_1810530_SM2463.zip › Supplementary information/Supplemental Informations.docx]

**Supplemental Figure 1:** Quality of life


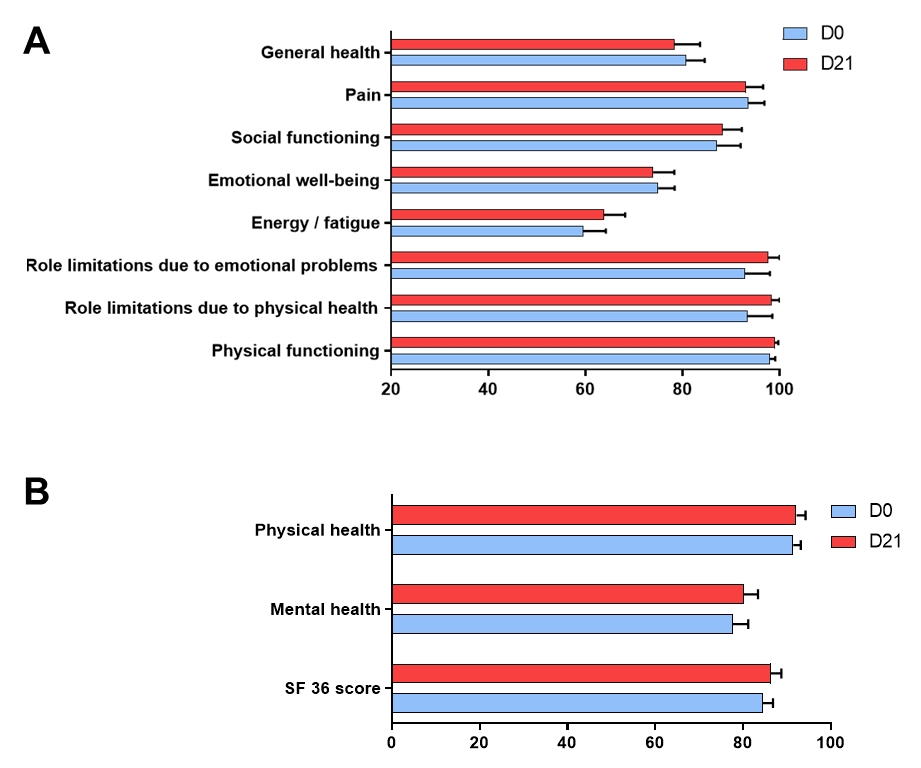


**A:** Eight domain scores of the 36-item short form survey scores. **B:** Domain scores collapsed to physical component summary “Physical health” or mental component summary “Mental health”.

**Supplemental Table 1:** Mean percentage of relative abundance of genera significantly different after CG intervention, 30 days before the intervention.

|  | **D-30** | **D0** | **p-value < 0.5** | **q-value <0.1** |
| --- | --- | --- | --- | --- |
| ***Genera*** |  | |  |  |
| *Bergeyella* | 0.78 ± 0.347 | 1.129 ± 0.425 | 0.033 | ns |
| *Blautia* | 5.315 ± 0.637 | 5.819 ± 0.533 | ns | ns |
| *Dorea* | 1.707 ± 0.175 | 2.228 ± 0.146 | 0.008 | ns |
| *Lachnospiraceae.UCG.004* | 0.175 ± 0.069 | 0.094 ± 0.032 | ns | ns |
| *Roseburia* | 1.404 ± 0.317 | 1.526 ± 0.316 | ns | ns |
| *Eubacterium* | 4.245 ± 0.473 | 4.673 ± 0.351 | ns | ns |
| *Ruminococcaceae.UCG.003* | 0.371 ± 0.131 | 0.252 ± 0.077 | ns | ns |
| *Ruminococcaceae.UCG.005* | 0.686 ± 0.16 | 0.74 ± 0.166 | ns | ns |
| *Subdoligranulum* | 1.621 ± 0.263 | 1.614 ± 0.24 | ns | ns |

A comparison of taxa significantly altered after 3 weeks of CG supplementation was made between the baseline (D0) and thirty days before the intervention (D-30, *n=13*). Results are expressed as mean percentage of relative abundance. Data are shown as mean ± SEM. Mean values are significantly different from baseline if p<0.05 (Wilcoxon matched-pairs test p<0.05, q<0.05 (FDR correction)).
